# Early mobilization in surgery: a narrative historical review of its evolution and contemporary implications

**DOI:** 10.1097/MS9.0000000000005698

**Published:** 2026-09-09

**Authors:** Martina Kostner, Federica Canzan, Elisa Ambrosi, Anna Brugnolli

**Affiliations:** a Department of Biomedicine and Prevention, University of Rome Tor Vergata, Roma, Italy; b Department of Diagnostics and Public Health, University of Verona, Verona, Italy; c Center for Medical Sciences (CISMed), University of Trento, Trento, Italy

**Keywords:** early mobilization, early mobilization history, postoperative, surgery

## Abstract

Early mobilization after surgery is widely recognized as a key component of enhanced recovery after surgery (ERAS) protocols, improving patient outcomes and reducing hospital stays. However, the historical roots of this practice remain insufficiently explored. This study aimed to examine the historical evolution of early mobilization from the 19th century to the mid-20th century and to reflect on its relevance for contemporary perioperative care. This study was designed as a narrative historical review based on primary historical sources, including medical articles, editorials, and case reports. Nine historical studies focusing on abdominal and pelvic surgery were included in the thematic analysis. The review identified seminal contributions such as Emil Ries’ pioneering 1899 regimen and the subsequent, though intermittent, adoption of early mobilization by surgeons including Boldt and Leithauser. Initially met with resistance rooted in traditional beliefs about prolonged bed rest, early mobilization gained wider acceptance after the Second World War, as reports described reduced complications, shorter hospital stays, and improved patient well-being. Early concepts comparable to modern prehabilitation and patient engagement were also identified. This historical reconstruction suggests that early mobilization is not solely a recent innovation but part of a longer clinical trajectory. Understanding its development may help inform current surgical practice and contribute to clearer operational definitions within contemporary perioperative pathways.

## Introduction and aim

Early postoperative mobilization is a key component of contemporary perioperative care. In contemporary perioperative care, early mobilization generally refers to structured patient mobilization initiated on the day of surgery or within the first 24 postoperative hours, although definitions and implementation criteria remain heterogeneous across studies and clinical settings[1]. Over the last two decades, substantial evidence has shown that early mobilization reduces postoperative complications and supports improved recovery and shorter hospital stays[1]. It is currently considered one of the pillars of Enhanced Recovery After Surgery (ERAS) protocols, a global surgical quality improvement initiative^[^2,3^]^. As early as 1990, Kehlet *et al* highlighted the benefits of structured perioperative interventions – including early mobilization – in reducing postoperative complications and attenuating the physiological stress response to surgery^[^4,5^]^. These studies laid the foundation for the ERAS movement, culminating in the first ERAS consensus statement in 2005[6].

HIGHLIGHTS

- Early mobilization was discussed in the surgical literature as early as 1899.
- Historical studies described highly structured postoperative mobilization protocols.
- Several historical practices anticipated principles now incorporated into enhanced recovery after surgery pathways.
- Early mobilization gained acceptance as reports showed reduced complications and shorter hospital stays.
- Standardized definitions and implementation criteria for early mobilization are still lacking today.

The shift toward reduced surgical invasiveness and accelerated recovery has progressively transformed traditional perioperative care models, which historically favored prolonged bed rest[7].

Interestingly, debates on early postoperative mobilization had already emerged at the beginning of the 20th century.

This narrative historical review aims to examine primary sources related to early mobilization from the late 19th century to the mid-20th century. Through thematic analysis, the study seeks to trace how the concept was defined, debated, and implemented and to explore how these historical experiences may contribute to current discussions on protocol standardization and the role of early mobilization within contemporary ERAS pathways.

## Methods

Before the consolidation of evidence-based medicine and standardized research reporting, surgical knowledge was primarily disseminated through case reports, editorials, opinion pieces, and professional correspondence.

This study was conducted as a narrative historical review based on primary historical sources.

A literature search was conducted between March and April 2025 using PubMed, Google Scholar, and manual bibliographic tracing of references and commentaries associated with retrieved articles. Search terms included combinations of “*early mobilization*,” *“early ambulation*,” *“postoperative care*,” *“bed rest,*” *and “surgery*.”

Eligible publications were original articles, case series, editorials, and commentaries published in English before 1950 that specifically addressed postoperative early mobilization in surgical settings. Articles that focused primarily on surgical techniques rather than on postoperative mobilization, publications unrelated to the topic, non-English articles, and publications without accessible full text were excluded.

Many historical publications are not indexed in contemporary electronic databases; therefore, several sources were identified through citation tracking (snowballing) of relevant primary articles, manual bibliographic tracing, and consultation of physical library archives to obtain historical publications that were not available in digital format. Given the historical nature of the material and the reliance on citation tracking and manual retrieval, a formal preferred reporting items for systematic reviews and meta-analyses-based selection process was not applied. Overall, 25 publications were initially screened, and nine historical studies meeting the inclusion criteria were ultimately included in the thematic analysis.

## Brief chronological history

### 1899–1930

Only in the final years of the 19th century did a particular discussion of early postoperative mobilization appear. In what can be considered a seminal article, Dr. Emil Ries, professor of gynecology at the Postgraduate Medical School in Chicago, described his unconventional postoperative regimen in 1899[8]. The article was likely read by gynecologists who adopted Ries’ practice in their own centers[7].

Ries not only challenged the customary 2–3-week hospital bed rest but also omitted the traditional preoperative routine of complete emptying of the intestinal tract. The regimen was largely based on clinical observation and practical reasoning. Although he believed that he was the first to implement such a plan extensively, he did not specify the number of patients treated. Importantly, he reassured his colleagues that no adverse reactions had been observed. In 1906, Dr. Boldt, who maintained contact with Ries, presented his positive experience with early mobilization after laparotomy at the annual conference of the Gynecological Surgery Society in Baltimore.

The following year, at the 1907 annual meeting of the American Gynecological Society, the advisability of early postoperative mobilization sparked a lengthy debate. Boldt presented a paper in favor, but several others opposed him, some dogmatically. For example, Dr. Baldy conceded that there was probably no benefit in keeping patients confined to bed for longer than 2 weeks. However, he firmly stated, *Prolonged absolute bed rest carries no dangers despite recent arbitrary claims by surgeons. Assertions that thrombosis, embolism, or phlebitis arise from prolonged recumbency are absurd and not supported by my own experience.”*[4] It took nearly 50 years before Emil Ries was widely recognized as a pioneer in this field. By then, his contributions had largely faded from collective memory and were remembered only by a few dedicated surgeons. Following his initial efforts, no further reports on early mobilization appeared, and it was neither widely practiced nor included in the academic curricula.

### 1930–1950

By the 1930s, surgery had become an increasingly established therapeutic option rather than a treatment of last resort. It had become safer, partly owing to improvements in asepsis and technical knowledge[7]. In 1930, Leithauser rediscovered this method. A historical anecdote states that, among the events that led Leithauser to focus on early mobilization, there were a few cases of patients who had disobeyed instructions to remain in bed after surgery and whose recovery had been surprisingly rapid. Leithauser and his associate, Howard L. Bergo, published their initial paper in the Archives of Surgery in 1941[9]. Leithauser subsequently published another case series, which was not without difficulty. He also described the reluctance of some publishers to disseminate recommendations considered excessively radical at the time. Acceptance of the practice of early mobilization grew considerably between 1945 and 1950. After the Second World War, numerous studies were published in favor of early mobilization, partly because it reduced pressure on hospital facilities and the burden of care.

## Thematic analysis

Nine historical studies were included in the thematic analysis. Their main characteristics, including study populations, types of surgery, and early mobilization procedures, are summarized in Table 1.

**Table 1:** Early mobilization protocols – historical studies (1899–1948).

Author, year, source	Population/field	Timing of mobilization	Detailed mobilization protocol
Ries, 1899 JAMA Surgery[8]	Number not reported: Women after vaginal celiotomy	POD 1	Out of bed within 24–48 h; muscle exercises and turning in bed encouraged immediately post-op.
Leithauser, 1941 Archives of Surgery[9]	383 patients appendectomy	POD 1	To get out of bed and standing bedside, take deep breathing exercises at regular intervals.
			Walk around the room and sit in a chair for a few moments.
Leithauser, 1943, Archives of Surgery[10]	464 patients abdominal surgery	POD 0	Patients were instructed on the day of operation to take deep breathing exercises and were assisted, if necessary, in changing position every hour.
			Technique: the morning after operation patients were requested to assume right lateral position, and were assisted to a sitting position. Bedside and cough. Respiratory exercises performed multiple times per day.
			Ambulation was gradually instituted.
Burch and Fisher, 1946 Annals of Surgery[11]	2827 veterans – hernia/abdominal surgery	POD 1	Early and active ambulation within the first 24 hours, under direct supervision or personal care.
Pocock and Kark, 1947 South African Medical Journal[12]	629 patients treated for appendectomy, cholecystectomy, hernia, or gastric/duodenal surgery	POD 0	To get out of bed within the first 24 to 48 hours.
			Increase the activity, walking around the bed, and rapid progress to regular ambulatory activity.
			POD 0: The patient is turned onto his side, usually on the side of incision, knees and thighs are flexed; he is raised sideways into a sitting position. He is assisted to a standing position. Deep breathing and coughing are encouraged after a few minutes, back into bed. Movement in bed is encouraged.
			POD1: in addition to standing, the patient walks some dozen steps before returning to bed. Then later walks and sits on a chair.
			Sympathetic reassurance and encouragement by the staff are important adjuncts to the technique.
Malloy, 1947 Journal of the National Medical Association[13]	160 patients gynecologic and major abdominal surgery	Within 72 h	Early rising: out of bed within the first 72 hours (better if 24 or 48).
Greenberg, 1948, Journal of the Medical Association of Georgia[14]	4 cases (abdominal surgery)	POD 1	Out of bed 24 hours after the operation.
Patterson and Richardson, 1948 North Carolina Medical Journal[15]	594 patients: 223 appendectomy, 120 hysterectomy, 105 pelvic surgery	POD 1	Detailed instructions on how to assist the patient in getting out of bed.
			Major operation: head of bed elevated, and then assistance to dangle his feet.
			Minor operation: sit up on the edge of the bed with only slight assistance.
			POD1: walk 10–20 paces with assistance, chair for 15–20 min (repeated 3 times).
			POD2: short walk in corridors and bathrooms. In-bed exercises: deep breathing, change position once an hour, dorsiflex the feet 1000 times/day.
Glickman *et al*, 1948 Archives of Physical Medicine[16]	51 patients age 16–66 herniorrhaphy	POD 1	Patients were required to report to the hospital 6 or 7 days before the operation. This period was devoted to stabilizing their diets, conducting tests, and performing prescribed exercises – calisthenics twice daily, both in and out of bed, timed for each muscle group.
			POD1: out of bed (3 min) and walking 1 min) and exercises: calisthenics (9 min in the morning and 11 min in the afternoon).
			POD2: exercises (calisthenics same vigor as preoperatively), walk short distance, out of bed 30 min.
			POD 3/4: out of bed 2–4 hours.

### Early mobilization practices

Although developed across different time periods and clinical settings, these contributions provide detailed documentation of early postoperative mobilization practices, well before the formalization of ERAS protocols. The surgical procedures described ranged from general abdominal surgery to gynecologic procedures, including appendectomy, hysterectomy, hernia repair, cholecystectomy, and other primary abdominal operations. Seven of the included studies reported sample sizes ranging from 51 to 2827 patients. In particular, studies by Burch and Fisher[11], Pocock and Kark[12], and Patterson and Richardson[15] each included more than 500 patients undergoing abdominal surgery, suggesting that early mobilization was applied systematically even in complex clinical contexts.

As early as 1899, Ries^[^8^]^ described encouraging patients who had undergone vaginal celiotomy to change position in bed and get out of bed within 2 days of surgery. In later studies, including those by Leithauser^[^9,10^]^ and Glickman *et al*[16], multimodal approaches were described. Patients were encouraged to perform deep-breathing exercises several times daily, change position hourly, sit up with assistance, and gradually begin ambulation. Glickman *et al*[16] also recommended foot dorsiflexion up to 1000 times per day, in addition to breathing exercises and positional changes.

Early mobilization protocols varied in their specific procedures but shared the intention of mobilizing patients within the first 24–48 hours following surgery. Most studies defined mobilization within the first 24 hours as “early,” while others extended this timeframe to 48 hours^[^8,12,13^]^. Leithauser^[^9,10^]^ recommended beginning mobilization at the bedside using a structured technique, followed by walking within the room and sitting in a chair for short periods. Other authors prescribed walking as early as the first postoperative day – for example, at least a dozen steps[12], or 10–20 steps followed by 15–20 minutes seated in a chair, repeated three times daily[15]. One study also included structured calisthenics sessions lasting 9 minutes in the morning and 11 minutes in the afternoon[16].

Most studies provided detailed descriptions of mobilization during the first postoperative day and reported progressive increases in walking distance and time spent out of bed in the subsequent days, including corridor ambulation and bathroom use from the second postoperative day onward^[^15,16^]^. One study defined delayed or standard mobilization as beginning on the eighth postoperative day[13]. However, several reports did not specify the duration of bed rest in standard practice, reflecting variability in perioperative routines at the time.

Nurses were frequently responsible for assessing patients’ tolerance for mobilization and for providing both practical and psychological support.

Several studies reported beneficial effects of early mobilization in older adults. Advanced age was not considered a contraindication, and in some cases, elderly patients expressed positive perceptions of their mobility and well-being^[^9,12,14^]^. Absolute contraindications commonly cited included shock, peritonitis, unstable wounds, and hemorrhage^[^9,11,13^]^.

### Advantages of early mobilization and measured outcomes

The approaches described involved progressive and graded movements, beginning with assisted sitting, followed by short walks within the room or along hospital corridors. Despite variations in specific protocols, most historical reports described beneficial effects associated with early mobilization, including reduced respiratory complications, improved patient well-being, and enhanced functional recovery. Several reports suggested that early mobilization may contribute to reductions in postoperative morbidity, particularly respiratory, thromboembolic, and cardiovascular complications^[^9–13,15^]^.

Improved patient mood was also noted in mobilized groups^[^11,12,15,16^]^, with some reports suggesting a reduced need for sedation[16]. Shorter hospital stays were frequently described, along with earlier return of bowel function, appetite, and vital capacity^[^10,11,13,16^]^. Although concerns were raised regarding wound dehiscence and incisional hernia, these complications were not observed in the reported series^[^10,11^]^.

### Early approaches to prehabilitation

In two studies, Leithauser[10] and Glickman and Keeton[16] described a preoperative phase approximately 1 week before surgery, aimed at functional preparation to improve postoperative recovery. This phase included respiratory and physical exercises as well as dietary optimization, anticipating principles that resemble those of contemporary prehabilitation strategies.

Several reports also emphasized patient involvement. Patients were informed about postoperative expectations and instructed to learn and repeat prescribed exercises^[^10,15,16^]^. Empathetic encouragement from healthcare professionals was considered essential, particularly in addressing patients’ concerns or reluctance regarding early mobilization^[^12,15^]^.

## Discussion

This narrative historical review analyzed primary historical documents concerning early mobilization between 1890 and the mid-20th century. The chronological reconstruction indicates that the term was first used by Ries^[8]^. In the following decades, early mobilization received limited attention until the 1940s. After the Second World War, however, the topic gained broader visibility in both clinical practice and surgical publications.

Contemporary commentaries reveal substantial resistance to abandoning prolonged bed rest. Statements such as Dr. Bonifield’s, “*Few of us doubted that. The experiences of ages are not to be thrust aside in a minute”*^[8]^, illustrate the strength of traditional beliefs regarding postoperative immobility. At the same time, other clinicians reported favorable experiences and emphasized the need to define mobilization more precisely, distinguishing structured ambulation from generic exercise[16].

Most historical reports described early mobilization protocols in considerable detail, including specific positioning techniques, timing, and repetitions. These descriptions suggest an effort to standardize postoperative movement within the constraints of surgical safety at the time. Although early proponents relied largely on empirical observation, some studies attempted to conduct more systematic comparative assessments.

Notably, several themes identified in these historical documents remain central to contemporary perioperative care. Outcomes such as recovery of bowel function, length of hospital stay, and reductions in respiratory and thromboembolic complications are still key endpoints in modern ERAS pathways. Furthermore, attention to patient cooperation and engagement had already been recognized as important for achieving clinical results. Several historical practices identified in this review, including ambulation within 24 hours, respiratory exercises, progressive mobilization, and patient engagement, anticipated concepts that are now incorporated into contemporary perioperative care and formal ERAS pathways. This continuity suggests that many principles underlying contemporary perioperative care were already being explored decades before the formalization of enhanced recovery programs. To facilitate interpretation of these findings, Figure 1 provides a chronological overview of the major historical milestones in the evolution of early postoperative mobilization and illustrates how several concepts progressively anticipated aspects of contemporary perioperative care, including elements now reflected in ERAS pathways. These associations are intended to highlight conceptual continuity rather than direct historical equivalence. When interpreting these historical reports, the concept of early mobilization should be understood within the clinical context of each period rather than according to a fixed contemporary time threshold. Although the specific timing of mobilization varied across historical reports, these interventions generally represented an intentional departure from the prevailing practice of prolonged postoperative bed rest. Accordingly, the parallels drawn with contemporary ERAS protocols are based not only on this shared clinical principle but also on the observation that several historical reports advocated ambulation within the first postoperative day, despite differences in terminology and clinical context.

**Figure 1.**
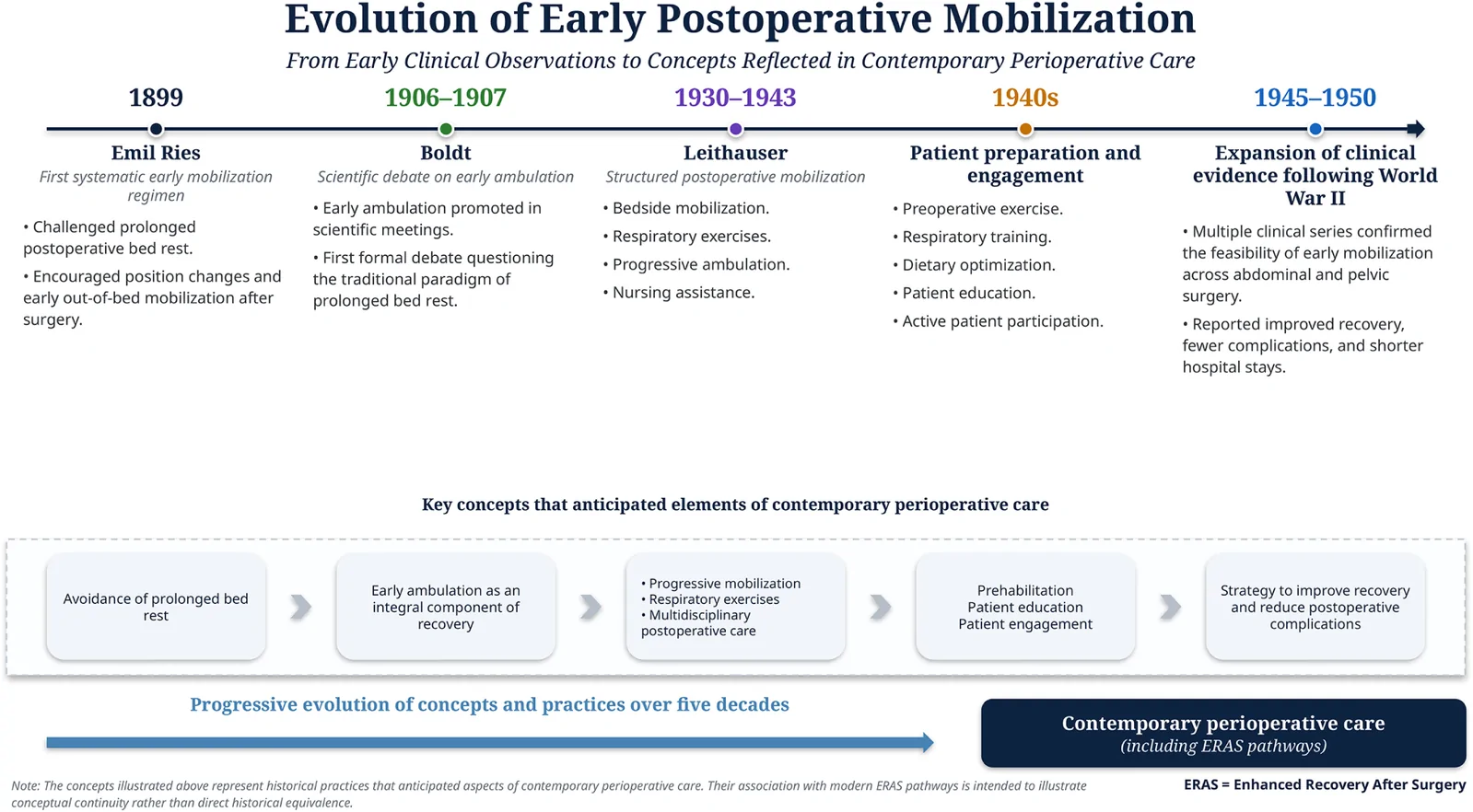
Evolution of early postoperative mobilization: historical milestones and concepts anticipating contemporary perioperative care.

Interestingly, several historical reports described postoperative mobilization protocols in highly detailed and prescriptive terms, including the number of steps, duration of ambulation, respiratory exercises, and time spent out of bed. In contrast, although contemporary ERAS guidelines strongly recommend early mobilization, generally beginning on the day of surgery or within the first 24 postoperative hours, specific operational protocols remain heterogeneous across studies and surgical settings. Recent ERAS recommendations for major abdominal and colorectal surgery[2] emphasize early ambulation and progressive mobilization for at least three hours daily from the first postoperative day onward, despite the low-to-moderate quality of evidence supporting some implementation criteria. This apparent paradox may reflect the evolution from early pragmatic clinical observations toward modern evidence synthesis based on heterogeneous primary studies using variable mobilization protocols. The continuity between historical and contemporary priorities suggests that early mobilization represents not a sudden innovation but an evolving clinical practice shaped by ongoing attempts to balance safety, recovery, and resource management. These findings may support ongoing efforts to standardize early mobilization protocols within ERAS pathways and reinforce its role as a measurable component of postoperative quality improvement.

## Limitations

This review has several limitations. The historical nature of the available sources limits reproducibility and introduces potential selection bias. Moreover, the historical record is inevitably incomplete, as some publications may have been lost, remained inaccessible, or were never widely disseminated. Historical literature may also be affected by reporting bias, with innovative approaches and favorable surgical outcomes being more likely to have been documented and preserved. Most of the included publications consisted of case series, editorials, and observational reports without standardized methodology or controlled comparisons. Furthermore, many historical articles lacked detailed outcome reporting, making direct comparisons between studies difficult. Consequently, the findings should be interpreted primarily as a historical and conceptual reconstruction rather than a quantitative evidence synthesis.

## Conclusion

This review demonstrates that early mobilization has well-established historical roots, first appearing in the surgical literature at the end of the 19th century. Although often perceived as a recent innovation associated with ERAS pathways, its fundamental principles were articulated decades earlier.

Over time, advances in physiological understanding have provided stronger explanations for benefits that early clinicians had already observed in practice. Interestingly, historical protocols were frequently detailed and prescriptive, reflecting structured attempts to regulate postoperative recovery.

Despite its widespread incorporation into contemporary perioperative care, early mobilization still lacks a universally accepted definition and standardized implementation criteria. At the same time, the surgical population has evolved, with increasing age and comorbidity burdens, highlighting the need for clearer operational frameworks and patient-centered strategies.

Revisiting the historical development of early mobilization provides perspective on its clinical evolution and may contribute to refining its application within modern surgical practice.

## Data Availability

No new data were generated or analysed in this study. All information discussed in this narrative historical review was derived from the published literature cited in the article.
